# Factors associated with university dropout intention among health sciences students: the mediating role of academic burnout and satisfaction with education

**DOI:** 10.1186/s12909-026-08973-7

**Published:** 2026-03-09

**Authors:** Laura Melisa Candelo-Meneses, Melany Andrea Castro-Velasco, Carlos Alberto Henao Periañez, Marcio Alexander Castillo-Díaz

**Affiliations:** 1 Facultad de Salud, Escuela de Enfermería, Programa de Enfermería, Universidad del Valle, Cali, Valle del Cauca Colombia; 2 Facultad de Salud, Escuela de Enfermería, Universidad del Valle, Cali, Valle del Cauca Colombia; 3 Departamento de Psicología/Maestría en Psicometría y Evaluación Educativa, Facultad de Ciencias Sociales, Universidad Nacional Autónoma de Honduras, Tegucigalpa, Honduras

**Keywords:** University dropout intention, Structural equation model, Health sciences students, Student burnout, Student Satisfaction

## Abstract

**Background:**

University dropout in health sciences programs threatens the stability of the future healthcare workforce, particularly in Latin American public universities facing structural and institutional constraints. Dropout intention is a strong predictor of actual dropout and reflects the interaction of personal, relational, and institutional factors. However, integrative models that examine these factors simultaneously and their mediating mechanisms remain limited. This study aimed to evaluate an explanatory model of university dropout intention among health sciences students, considering the mediating role of academic burnout and satisfaction with education.

**Methods:**

A cross-sectional observational study was conducted between March and September 2025 among undergraduate health sciences students at a public university in Cali, Colombia. A proportionate stratified random sample was obtained. Data were collected using validated self-report instruments assessing personal, relational, and institutional risk factors, academic burnout, satisfaction with education, and university dropout intention. Structural equation modeling was applied using the weighted least squares mean and variance adjusted (WLSMV) estimator to examine direct and indirect (mediated) effects.

**Results:**

The sample comprised 320 undergraduate health sciences students (69.1% female; mean age = 22.46 ± 3.47 years). The final structural model demonstrated adequate fit and explained 86.3% of the variance in university dropout intention. Academic burnout emerged as the strongest predictor of dropout intention, whereas satisfaction with education showed a significant protective effect. Several personal, relational, and institutional factors were directly and indirectly associated with dropout intention. Dysfunctional support networks and vocational decision-making were positively associated with dropout intention directly and through academic burnout and satisfaction with education, whereas curricular support and functional support networks were negatively associated with dropout intention primarily through higher satisfaction with education.

**Conclusions:**

University dropout intention among health sciences students is a multidimensional phenomenon mediated by personal, relational, and institutional factors. Interventions aimed at enhancing satisfaction with education and preventing academic burnout are essential to strengthen student retention and well-being in health sciences education.

**Supplementary Information:**

The online version contains supplementary material available at 10.1186/s12909-026-08973-7.

## Introduction

University dropout constitutes a major challenge for higher education systems due to its academic, economic, and social implications for both institutions and students [1, 2]. In the health sciences, this phenomenon is particularly relevant, given the impact that interruptions in training have on the availability and quality of the human talent required to meet the needs of health systems [3].

Dropout intention represents a significant concern among health sciences students, largely due to the high academic workload and the emotional demands inherent to these training programs [4]. Recent cross-sectional quantitative studies indicate a high prevalence of dropout intention among medical and nursing students, associated with academic stress and psychological distress [5–7]. Within this population, academic burnout has been identified as a direct predictor of dropout intention, whereas academic engagement, institutional support, and mentoring support function as protective factors that promote student retention in higher education [8–10].

Recent quantitative and longitudinal research has consolidated dropout intention as a valid and predictive indicator of actual university dropout, enabling the development of explanatory models aimed at identifying associated factors and underlying mechanisms in the dropout process [11]. These models converge in conceptualizing dropout as a multicausal phenomenon in which individual, relational, and institutional factors interact [2]. One of the theoretical frameworks supporting this integrative approach is the ecological theory of human development, which posits that individuals’ behavior and trajectories are shaped through the dynamic interaction between personal characteristics and multiple interrelated contextual systems [12].

When applied to the university context, this perspective makes it possible to understand dropout not as an exclusively individual phenomenon, but rather as the result of the interaction among personal, relational, and institutional factors operating simultaneously and cumulatively. In line with this approach, explanatory models have been proposed to assess the risk of university dropout by organizing associated factors across micro-, meso-, and macro-levels, which has facilitated their empirical examination in studies on student retention and dropout [13, 14].

At the individual or micro level, explanatory models, primarily based on quantitative survey studies and structural equation modeling analyses, have identified cognitive, affective, and motivational variables associated with dropout intention. Academic self-efficacy, perceived academic control, and academic engagement have been linked to a lower likelihood of dropout intention, whereas academic stress, anxiety, depression, and academic burnout are associated with an increased risk of dropout [9, 15, 16].

At the relational (meso) level, empirical evidence highlights the role of family, peers, and institutional support networks in student retention. Studies conducted across different cultural contexts show that higher perceived social support is associated with lower levels of dropout intention, whereas the presence of conflictual relationships, social isolation, or negative relational climates increases the risk of dropout [15, 17]. These findings suggest that immediate relational systems may buffer or exacerbate the impact of academic demands on students’ trajectories [9].

Finally, at the institutional or macro level, a substantial body of research has documented the influence of structural factors within the educational system on university dropout intention. Curricular organization, perceived teaching quality, availability of academic resources, and alignment between educational training and expectations regarding labor market integration have been incorporated into explanatory and predictive models of dropout [2, 10]. In addition, qualitative studies have shown that negative perceptions of the institutional environment and of future opportunities associated with university education contribute to dropout intention, particularly when they coexist with high academic demands and low levels of institutional support [18].

From an ecological perspective, these factors can be understood as conditions that shape students’ university experiences and influence their persistence within the system. However, most studies have focused on examining direct associations between these factors and dropout, paying comparatively less attention to the mechanisms through which such factors exert their influence.

In this context, academic burnout has emerged as a key construct for understanding the processes leading to dropout intention. Academic burnout is characterized by a state of emotional and cognitive exhaustion in response to persistent academic demands and has been consistently associated with poor academic performance, psychological distress, and a higher likelihood of dropout [4]. Cross-sectional studies have shown a high prevalence of academic burnout among university students, particularly in health-related programs, linked to academic overload, emotional demands, and highly demanding training conditions [4, 19]. Among nursing and medical students, higher levels of burnout are directly associated with greater psychological distress and a stronger intention to abandon their studies [5, 7]. Recent research has provided empirical evidence positioning academic burnout as an intermediate factor between contextual conditions and dropout intention, showing that variables such as peer learning or academic engagement influence dropout intention primarily through their effect on burnout [20, 21]. Furthermore, longitudinal and prospective studies in students enrolled in health programs indicate that sustained exposure to adverse physical and psychological conditions, together with higher levels of academic burnout, increases the likelihood of dropout intention and actual dropout [20, 22].

Complementarily, satisfaction with education has been identified as a protective factor against university dropout. A recent systematic review concluded that dissatisfaction with the university experience is significantly associated with a higher likelihood of dropout, and that satisfaction operates as an intermediate process explaining how various academic and institutional conditions influence the decision to leave higher education [23]. Recent quantitative mediation studies show that satisfaction is negatively related to dropout intention and mediates the effect of relevant educational variables—such as academic engagement and the use of self-regulation strategies—on student retention [24]. From an institutional perspective, quantitative institutional studies indicate that organizational identification and institutional reputation influence retention behaviors primarily through their impact on student satisfaction [25]. Likewise, quantitative research examining educational service quality confirms that these variables are positively associated with student satisfaction, which in turn is linked to higher levels of loyalty and lower dropout intention [26, 27].

Despite advances in understanding the factors associated with university dropout, a gap remains in the literature regarding integrative models that simultaneously consider personal, relational, and institutional factors and evaluate the mediating role of academic burnout and satisfaction with education in dropout intention, particularly among health sciences students. In Latin America, the structural characteristics of public universities, socioeconomic inequalities, and limitations within educational systems shape contexts that differentially influence university dropout intention [1]. Most explanatory models of university dropout have been developed and tested in high-income countries, where institutional resources, student support systems, and socioeconomic conditions differ substantially from those in many Latin American public universities. In contrast, higher levels of socioeconomic inequality, constrained institutional funding, and a greater proportion of students who are the first in their families to access higher education may intensify academic strain and modify the mechanisms through which burnout and satisfaction influence dropout intention.

Therefore, the availability of contextualized evidence is essential for understanding the interaction of personal, relational, and institutional factors and for identifying relevant mediating mechanisms, such as academic burnout and satisfaction with education. The development of explanatory models based on local data helps reduce gaps in the international literature and provides empirical inputs to inform student retention strategies and institutional decision-making aligned with the realities of higher education in the region.

In response to this gap, the present study aimed to evaluate an explanatory model of university dropout intention among students from a Faculty of Health, analyzing the direct and indirect effects of personal, relational, and institutional factors, while considering the mediating role of satisfaction with education and academic burnout.

**Fig. 1:**
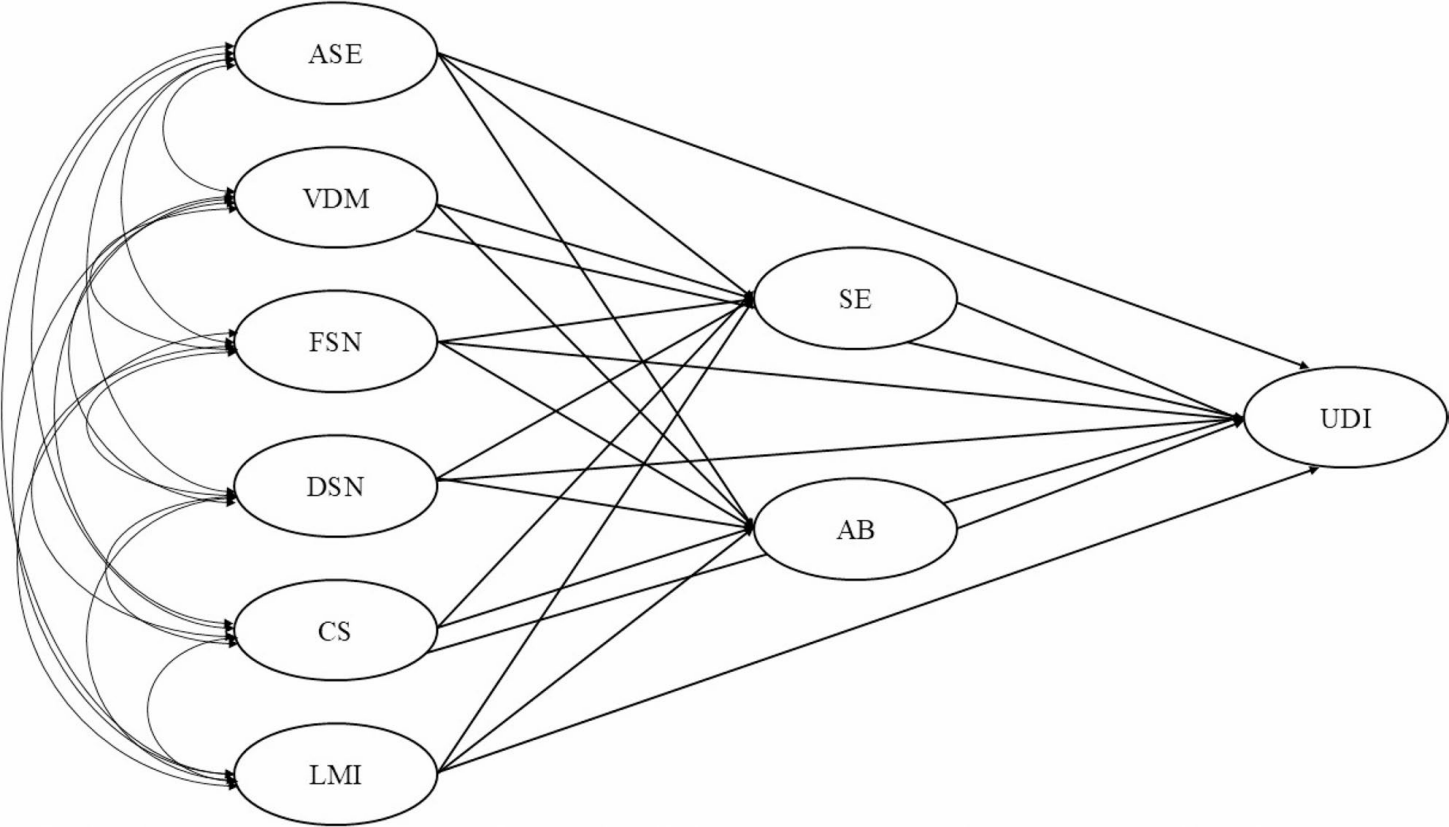
Hypothetical structural model of university dropout intention. ASE= Academic self-efficacy, VDM= Vocational decision-making, FSN= Functional support networks, DSN= Dysfunctional support networks, CS= Curricular support, LMI= Labor market integration, AB= Academic Burnout, SE= Satisfaction with Education, UDI= University Dropout Intention

Based on this objective, Fig. 1 presents the proposed hypothetical structural model to examine the factors associated with university dropout intention among health sciences students. In this model, risk factors are conceptualized across three interrelated levels: personal, relational, and institutional. Personal factors include self-efficacy and vocational decision-making, understood respectively as students’ perceived confidence in their ability to successfully manage academic demands and the degree of clarity and commitment regarding their chosen field of study. Relational factors comprise functional and dysfunctional support networks, referring to the perceived availability of supportive relationships (family, peers, faculty) and the presence of conflictual or unsupportive interactions within students’ immediate social environment. Institutional factors encompass curricular support and labor market integration, defined as students’ perceptions of academic guidance, curricular organization, and the extent to which their training aligns with future professional opportunities [13, 14].

Drawing on prior evidence, these dimensions are hypothesized to be directly associated with university dropout intention. In addition, personal, relational, and institutional risk factors are expected to be directly related to academic burnout and satisfaction with education.

Regarding indirect effects, academic burnout and satisfaction with education are proposed to act as mediating variables in the association between risk factors and university dropout intention. Accordingly, the following hypotheses are formulated:

- H1: Personal (self-efficacy and vocational decision-making), relational (functional and dysfunctional support networks), and institutional (curricular support and labor market integration) risk factors are directly associated with university dropout intention (UDI).
- H2: Personal, relational, and institutional risk factors are directly associated with academic burnout (AB) and satisfaction with education (SE).
- H3: Academic burnout (AB) and satisfaction with education (SE) mediate the association between personal, relational, and institutional risk factors and university dropout intention (UDI).

The proposed model allows for an integrated examination of the direct and indirect effects of multiple dimensions of the academic and psychosocial environment on university dropout intention (UDI), providing a robust analytical framework for the design of institutional interventions aimed at preventing university dropout among health sciences students.

## Method

An observational cross-sectional study was conducted between March and September 2025, guided by the recommendations of the STROBE (Strengthening the Reporting of Observational Studies in Epidemiology) initiative for observational studies [28].

### Population, sample, and sampling

The study was carried out at the Faculty of Health of a public university located in the city of Cali, in southwestern Colombia. This faculty offers academic programs at the technological, undergraduate, and postgraduate levels in the field of health sciences. During the 2024–2 academic period, the population of students enrolled in undergraduate programs comprised 1,858 students, who constituted the target population for the present study.

The sample size was calculated based on an approximate population of 1,858 undergraduate students, assuming an expected prevalence of 50%, a 95% confidence level, and a maximum acceptable margin of error of 5%. Under these assumptions, a minimum required sample size of 319 participants was determined.

A proportionate stratified sampling design was employed according to academic program to ensure representativeness across groups. The strata corresponded to the programs of Medicine, Nursing, Dentistry, Physiotherapy, Occupational Therapy, Speech and Language Therapy, and Bacteriology. Sample allocation within each stratum was proportional to the size of each program within the total population. Within each stratum, participants were selected using simple random sampling.

### Inclusion criteria

Students aged 18 years or older who were enrolled in undergraduate programs at the Faculty of Health during the 2025–1 and 2025–2 academic periods and who voluntarily agreed to participate and provided informed consent were included.

### Exclusion criteria

Students who did not agree to participate, did not provide informed consent, or did not complete the questionnaire in its entirety were excluded.

### Data collection instruments and techniques

Data were collected using a structured, self-administered questionnaire delivered electronically via Google Forms. The instrument included sociodemographic and academic variables, university dropout risk, and university dropout intention.

Dropout risk factors were assessed using the University Dropout Questionnaire for Students (CDUe) [13], which is based on the Ecological Theory of Human Development [12]. The CDUe consists of 63 items distributed across six dimensions: academic self-efficacy (ASE), vocational decision-making (VDM), functional support networks (FSN), dysfunctional support networks (DSN), curricular support (CS), and labor market integration (LMI). with responses recorded on a five-point Likert scale (0 = disagree to 4 = strongly agree). Overall, higher scores reflect greater dropout risk. Specifically, higher scores on academic self-efficacy (ASE), vocational decision-making (VDM), dysfunctional support networks (DSN), curricular support (CS), and labor market integration (LMI) indicate greater perceived academic, vocational, relational, and institutional difficulties associated with dropout risk, whereas higher scores on the functional support networks (FSN) dimension indicate greater perceived availability of functional academic and social support. The instrument has demonstrated prior evidence of construct and convergent validity, as well as adequate internal consistency (α = 0.68; ω = 0.67), and has been validated in samples of Colombian university students [13].

University dropout intention (UDI), satisfaction with education (SE), and academic burnout (AB) were assessed using the Screening Instrument for Students At-Risk of Dropping Out [29], a self-report measure grounded in a multidimensional theoretical model of academic dropout. This instrument comprises three dimensions— university dropout intention (UDI), satisfaction with education (SE), and academic burnout (AB)—each consisting of four items. Items are rated on a five-point Likert scale (1 = strongly disagree to 5 = strongly agree). The instrument has demonstrated evidence of construct validity, and its subscales showed adequate internal consistency: academic burnout (AB) (α = 0.83; ω = 0.83), satisfaction with education (SE) (α = 0.81; ω = 0.80), and university dropout intention (UDI) (α = 0.76; ω = 0.75) [29]. For all subscales, higher scores indicate higher levels of the assessed construct.

Before accessing the questionnaire, students were presented with a digital informed consent form, acceptance of which was required to proceed. Participation was voluntary and anonymous, with an estimated completion time of 15 to 20 min, ensuring the confidentiality of the information. Upon completion of the survey, participants were provided with information about the student support programs offered by the university, aimed at academic and psychosocial support and overall well-being.

### Ethical considerations

The study was reviewed and approved by the Research Ethics Committee of Universidad del Valle, Cali, Colombia (Record No. 093 − 024, November 6, 2024). All participants provided voluntary informed consent prior to inclusion in the study. Clear information regarding the study objectives was provided, participation was stated to involve no foreseeable risks, and participants were assured of their right to withdraw from the study at any time without academic or any other consequences. No ethical concerns or adverse events were reported during the data collection process.

### Data analysis

Data analysis was conducted in three sequential phases. The first phase involved the generation of descriptive statistics for all study variables. Categorical variables are presented as frequencies and percentages. Quantitative variables are reported using means, standard deviations, minimum and maximum values, histograms, skewness, and kurtosis.

Because all variables were assessed through self-report measures at a single time point, common method bias was examined using an exploratory factor analysis including all study items. The analysis yielded 12 factors with eigenvalues greater than 1, and the first factor accounted for 31.15% of the total variance. Following commonly cited criteria, values below 40% suggest that severe common method bias is unlikely [30].

In the second phase, bivariate analyses were performed for the main study variables. Prior to inferential analyses, the assumption of normality was assessed using the Shapiro–Wilk test. Given that the results indicated significant departures from normality in several of the analyzed variables, Spearman’s rank correlation coefficient was used, as it is more robust to asymmetric distributions and the presence of outliers.

The third phase of the analysis involved hypothesis testing. In this phase, a structural equation modeling (SEM) approach was employed, which simultaneously integrates measurement models—where latent variables account for their respective observed indicators (items)—and the structural model, which estimates direct and indirect relationships among latent constructs [31]. The measurement models for each instrument were previously evaluated using confirmatory factor analysis at the item level. The reliability of each factor was assessed using ordinal alpha and McDonald’s omega coefficients. Values above 0.70 were considered indicative of adequate reliability [32]. Likewise, average variance extracted (AVE) is reported as an indicator of precision, with values above 0.50 regarded as acceptable [33]. Discriminant validity among latent constructs was additionally evaluated using the heterotrait–monotrait ratio of correlations (HTMT), applying the conservative threshold of 0.85 as recommended by Henseler et al. [34].

The specified structural model corresponds to a multiple mediation framework in which personal, relational, and institutional risk factors predict dropout intention both directly and indirectly through satisfaction with education (SE) and academic burnout (AB) (see Fig. 1). Model estimation was conducted using the weighted least squares mean and variance adjusted (WLSMV) estimator, which is appropriate for ordinal variables [31]. The fit of both the measurement models and the structural model was evaluated using the comparative fit index (CFI) and the Tucker–Lewis’s index (TLI), with values ≥ 0.95 considered indicative of good fit [35]. Likewise, the root mean square error of approximation (RMSEA) and the standardized root mean square residual (SRMR) were examined, with values ≤ 0.08 regarded as acceptable [36].

At this stage of the analysis, direct effects on each endogenous variable were examined, as well as specific indirect effects (through each mediator) and total effects (direct + indirect). All reported path coefficients, including indirect and total effects, correspond to fully standardized estimates (std.all in *lavaan*). To assess the overall explanatory capacity of the model, coefficients of determination (R²) were additionally reported for each endogenous variable. 95% confidence intervals for the structural effects were estimated using the delta method [37].

All analyses were conducted in R (version 4.4.2) using the RStudio environment. Descriptive analyses were performed with the *skimr* package (version 2.1.5) [38]. Estimation and evaluation of the measurement and structural models were carried out using the *lavaan* package (version 0.6–16) [39] and the *semTools* package (version 0.5–6) [40].

## Results

### Descriptive analyses

The sample comprised 320 undergraduate students, with a mean age of 22.46 ± 3.47 years, predominantly female (69.1%) and single (92.8%). Most participants belonged to low and middle socioeconomic strata (96.3%), were not employed (63.4%), and lived with their families (69.1%). Academically, students were enrolled in various health sciences programs, mainly Medicine, Dentistry, and Occupational Therapy, and the majority reported a grade point average between 4.0 and 5.0 (76.9%). The final distribution of participants by academic program reflected the proportional stratified sampling design, ensuring representation across all strata. The number and percentage of students in each academic program are presented in Table 1, confirming the representativeness of the sample relative to the target population. Sample characteristics are presented in Table 1.

**Table 1:** Socio-demographic and academic characteristics of undergraduate students in health sciences (*n* = 320)

Variable	*n* (%) / M ± SD
Age (years)	22.46 ± 3.47
Gender	
Female	221 (69.1)
Male	99 (30.9)
Marital status	
Single	297 (92.8)
Married or cohabiting	23 (7.2)
Monthly household income*	
< 2 minimum monthly wages	214 (66.9)
≥ 2 minimum monthly wages	106 (33.1)
Living arrangement	
With family (nuclear or extended)	221 (69.1)
Alone or with non-family members	99 (30.9)
Employment status	
Not employed	203 (63.4)
Employed	117 (36.6)
Academic program	
Bacteriology	38 (11.9)
Nursing	41 (12.8)
Physiotherapy	39 (12.2)
Speech and language therapy	39 (12.2)
Medicine	68 (21.3)
Dentistry	47 (14.7)
Occupational therapy	48 (15.0)
GPA	
3.0–3.9	71 (22.2)
4.0–5.0	246 (76.9)
< 3.0	3 (0.9)

*M* Mean, *SD* Standard deviation

*n* (%) = frequency and percentage

* = Minimum monthly wage refers to the legal minimum wage in Colombia; *GPA* Grade point average (range: 0.0–5.0)

Table 2 presents the descriptive statistics of the latent variables included in the study. The findings indicate that academic self-efficacy (ASE), functional support networks (FSN), curricular support (CS), and labor market integration (LMI) exhibited distributions centered around moderate values with relatively symmetric shapes, reflecting balanced scores within the sample. In contrast, satisfaction with education (SE) showed a marked concentration at higher values, whereas vocational decision-making (VDM), dysfunctional support networks (DSN), and university dropout intention (UDI) displayed distributions skewed toward lower values, indicating that scores were predominantly concentrated in the lower range of the scale. Academic burnout (AB) was observed at intermediate levels, slightly oriented toward moderate-to-low values.

**Table 2:** Descriptive statistics of personal, relational, and institutional factors and university dropout intention (*n* = 320)

Variables	Mean	SD	Min.	Max.	Histogram	Skewness	Kurtosis
ASE	17.80	9.49	0	48	▃▇▅▂▁	0.395	0.201
VDM	6.98	6.67	0	28	▇▅▂▁▁	1.016	0.452
FSN	26.00	7.83	0	40	▁▁▅▇▃	−0.550	0.933
DSN	6.82	6.50	0	32	▇▃▂▁▁	1.288	2.168
CS	19.60	10.24	0	48	▃▇▇▃▁	0.159	0.178
LMI	19.70	13.20	0	56	▇▇▇▂▁	0.390	−0.060
SE	16.60	3.82	4	20	▁▁▂▅▇	−1.428	1.828
AB	11.00	4.64	4	20	▇▇▇▅▅	0.261	−0.812
UDI	8.15	4.66	4	20	▇▃▂▁▁	1.155	0.384

*SD* Standard deviation, *ASE* Academic self-efficacy, *VDM* Vocational decision-making, *FSN* Functional support networks, *DSN* Dysfunctional support networks, *CS* Curricular support, *LMI* Labor market integration, *SE* Satisfaction with education, *AB* Academic burnout, *UDI* University dropout intention. Histogram symbols represent relative frequency distribution across score ranges

### Bivariate analyses

Table 3 presents the correlation coefficients among the study variables. Within the set of personal factors, academic self-efficacy (ASE) and vocational decision-making (VDM) showed a positive association with each other, and both were consistently related to institutional factors, particularly labor market integration (LMI) and curricular support (CS).

**Table 3:** Correlations among personal, relational, and institutional factors and university dropout intention (*n* = 320)

Variables	1	2	3	4	5	6	7	8	9
1. ASE	1								
2. VDM	0.407**	1							
3. FSN	−0.142*	−0.229**	1						
4. DSN	0.427**	0.410**	−0.216**	1					
5. CS	0.431**	0.375**	−0.108	0.458**	1				
6. LMI	0.504**	0.523**	−0.205**	0.412**	0.485**	1			
7. SE	−0.235**	−0.323**	0.435**	−0.354**	−0.163*	−0.259**	1		
8. AB	0.437**	0.307**	−0.074	0.344**	0.313**	0.429**	−0.150*	1	
9. UDI	0.260**	0.384**	−0.106	0.373**	0.170*	0.313**	−0.344*	0.618**	1

*ASE* Academic self-efficacy, *VDM* Vocational decision-making, *FSN* Functional support networks, *DSN* Dysfunctional support networks, *CS* Curricular support, *LMI* Labor market integration, *SE* Satisfaction with education, *AB* Academic burnout, *UDI* University dropout intention

* *p* < 0.05; ** *p* < 0.001

Among relational factors, functional support networks (FSN) were positively associated with satisfaction with education (SE) and negatively associated with dysfunctional support networks (DSN). In turn, dysfunctional support networks (DSN) showed positive correlations with academic burnout (AB) and university dropout intention (UDI).

Regarding institutional factors, both curricular support (CS) and labor market integration (LMI) were positively associated with academic self-efficacy (ASE) and vocational decision-making (VDM), and negatively associated with satisfaction with education (SE).

With respect to the academic experience, satisfaction with education (SE) showed negative associations with academic burnout (AB) and university dropout intention (UDI), whereas academic burnout (AB) exhibited the strongest correlation with university dropout intention (UDI) across the entire matrix. Finally, university dropout intention (UDI) was positively associated with dysfunctional support networks (DSN), academic burnout (AB), vocational decision-making (VDM), academic self-efficacy (ASE), and labor market integration (LMI), and negatively associated with satisfaction with education (SE).

### Structural equation modeling analysis

#### Measurement models

The measurement structure of the CDUe was evaluated using a confirmatory factor analysis specifying six first-order factors: academic self-efficacy (ASE), vocational decision-making (VDM), functional support networks (FSN), dysfunctional support networks (DSN), curricular support (CS), and labor market integration (LMI). The model demonstrated adequate fit to the data, supporting the proposed theoretical structure (CFI = 0.981; TLI = 0.980; SRMR = 0.078; RMSEA = 0.073, 95% CI = 0.070–0.075). Standardized factor loadings were statistically significant (*p* < 0.001) and ranged from λ = 0.537 to 0.829 for ASE, λ = 0.798 to 0.936 for VDM, λ = 0.519 to 0.930 for FSN, λ = 0.641 to 0.910 for DSN, λ = 0.600 to 0.800 for CS, and λ = 0.729 to 0.877 for LMI. Detailed item-level results, including standardized factor loadings, standard errors, p values, and residual variances, are provided in Supplementary Table S1. Reliability was high across all dimensions (ω = 0.897–0.953; α = 0.861–0.946). In addition, average variance extracted (AVE) values exceeded 0.50 for all factors (range = 0.520–0.785). All HTMT values were below the specified threshold, with a maximum value of 0.620, supporting adequate discriminant validity among the latent dimensions (see Supplementary Table S2).

For the Screening Instrument for Students At-Risk of Dropping Out, a three-factor first-order model corresponding to satisfaction with education (SE), academic burnout (AB), and university dropout intention (UDI) was estimated. The model showed excellent overall fit (CFI = 0.998; TLI = 0.998; SRMR = 0.058; RMSEA = 0.069, 95% CI = 0.054–0.084). Standardized factor loadings were high and statistically significant (*p* < 0.001), ranging from λ = 0.909 to 0.965 for satisfaction with education (SE), λ = 0.784 to 0.976 for university dropout intention (UDI), and λ = 0.761 to 0.930 for academic burnout (AB). Detailed item-level results, including standardized factor loadings, standard errors, p values, and residual variances, are provided in Supplementary Table S3. Reliability was high for all dimensions (ω = 0.901–0.938; α = 0.893–0.943), and AVE values were also high (0.745–0.870). HTMT values were likewise below the established cutoff (maximum = 0.825), indicating adequate discriminant validity among satisfaction with education (SE), academic burnout (AB), and university dropout intention (UDI) (see Supplementary Table S4).

#### Structural model

**Fig. 2:**
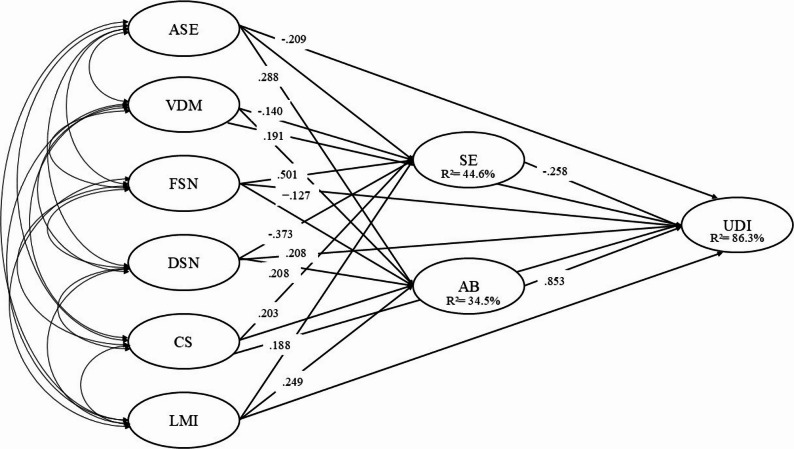
Final structural equation model with standardized path coefficients (β) and explained variance (R²). Notes. ASE = Academic self-efficacy; VDM = Vocational decision-making; FSN = Functional support networks; DSN = Dysfunctional support networks; CS = Curricular support; LMI = Labor market integration; SE = Satisfaction with education; AB = Academic burnout; UDI = University dropout intention. Values represent standardized regression coefficients (β). To enhance clarity, only statistically significant paths (p < 0.05) are displayed. R² values indicate the proportion of variance explained in endogenous variables.

The multivariate analysis of the structural model demonstrated good fit to the data (χ² = 6,822.818; df = 2,664; *p* < 0.001; CFI = 0.982; TLI = 0.981; SRMR = 0.077; RMSEA = 0.070 [90% CI = 0.068, 0.072]). Table 4 presents the results of the standardized direct effects of the CDUe instrument dimensions on university dropout intention (UDI), satisfaction with education (SE), and academic burnout (AB). The final structural model with standardized path coefficients is displayed in Fig. 2. Correlations among the exogenous latent variables are reported in Supplementary Table S5.

**Table 4:** Standardized direct effects of personal, relational, and institutional factors on university dropout intention (UDI), satisfaction with education (SE) and academic burnout (AB) (*n* = 320)

Predictor	Outcome	β	95% CI	*p*
ASE →	UDI	−0.209	−0.307, − 0.110	**< 0.001**
VDM →	UDI	0.191	0.084, 0.298	**< 0.001**
FSN →	UDI	−0.127	0.039, 0.215	**0.006**
DSN →	UDI	0.208	0.096, 0.319	**< 0.001**
CS →	UDI	−0.188	−0.291, − 0.084	**< 0.001**
LMI →	UDI	−0.039	−0.134, 0.056	0.419
SE →	UDI	−0.258	−0.362, − 0.154	**< 0.001**
AB →	UDI	0.853	0.784, 0.922	**< 0.001**
ASE →	SE	−0.048	−0.184, 0.087	0.484
VDM →	SE	−0.140	−0.272, − 0.009	**0.037**
FSN →	SE	0.501	0.423, 0.579	**< 0.001**
DSN →	SE	−0.373	−0.503, − 0.242	**< 0.001**
CS →	SE	−0.203	−0.077, − 0.330	**0.002**
LMI →	SE	0.063	−0.074, 0.201	0.365
ASE →	AB	0.288	0.175, 0.402	**< 0.001**
VDM →	AB	0.059	−0.079, 0.197	0.401
FSN →	AB	0.047	−0.059, 0.154	0.382
DSN →	AB	0.208	0.057, 0.358	**0.008**
CS →	AB	−0.071	−0.211, 0.068	0.318
LMI →	AB	0.249	0.118, 0.381	**< 0.001**

*ASE* Academic self-efficacy, *VDM* Vocational decision-making, *FSN* Functional support networks, *DSN* Dysfunctional support networks, *CS* Curricular support, *LMI* Labor market integration, *SE* Satisfaction with education, *AB* Academic burnout, *UDI* University dropout intention, *β* standardized regression coefficient, *CI* confidence interval

Bold values indicate statistically significant coefficients (*p* < .05)

With regard to university dropout intention (UDI), academic self-efficacy (ASE) (β = −0.209, 95% CI [− 0.307, − 0.110], *p* < 0.001), curricular support (CS) (β = −0.188, 95% CI [− 0.291, − 0.084], *p* < 0.001), and satisfaction with education (SE) (β = −0.258, 95% CI [− 0.362, − 0.154], *p* < 0.001) showed significant negative effects, indicating that higher levels of these dimensions are associated with lower university dropout intention (UDI).

In contrast, vocational decision-making (VDM) (β = 0.191, 95% CI [0.084, 0.298], *p* = 0.001), dysfunctional support networks (DSN) (β = 0.208, 95% CI [0.096, 0.319], *p* < 0.001), and, most notably, academic burnout (AB) (β = 0.853, 95% CI [0.784, 0.922], *p* < 0.001) exhibited significant positive effects on university dropout intention (UDI). Functional support networks (FSN) (β = −0.127, 95% CI [− 0.039, − 0.215], *p* = 0.006) showed a significant effect of smaller magnitude, whereas labor market integration (LMI) did not show a significant direct effect on this outcome (*p* = 0.419).

With respect to satisfaction with education (SE), functional support networks (FSN) (β = 0.501, 95% CI [0.423, 0.577], *p* < 0.001) and curricular support (CS) (β = 0.203, 95% CI [0.077, 0.330], *p* = 0.002) showed significant positive effects, whereas dysfunctional support networks (DSN) (β = −0.373, 95% CI [− 0.503, − 0.242], *p* < 0.001) and vocational decision-making (VDM) (β = −0.140, 95% CI [− 0.272, − 0.009], *p* = 0.037) exhibited significant negative effects. Academic self-efficacy (ASE) and labor market integration (LMI) did not show significant direct effects on satisfaction with education (SE) (*p* > 0.05).

Regarding academic burnout (AB), academic self-efficacy (ASE) (β = 0.288, 95% CI [0.175, 0.402], *p* < 0.001), dysfunctional support networks (DSN) (β = 0.208, 95% CI [0.057, 0.358], *p* = 0.008), and labor market integration (LMI) (β = 0.249, 95% CI [0.118, 0.381], *p* < 0.001) showed significant positive effects. In contrast, vocational decision-making (VDM), functional support networks (FSN), and curricular support (CS) did not present significant direct effects on academic burnout (AB) (*p* > 0.05).

Table 5 presents the standardized indirect effects of personal, relational, and institutional factors on university dropout intention (UDI) through satisfaction with education (SE) and academic burnout (AB), as well as the total effects (direct + indirect). Regarding mediations via satisfaction with education (SE), vocational decision-making (VDM) (β = 0.036, 95% CI [0.001, 0.071], *p* = 0.042), functional support networks (FSN) (β = −0.129, 95% CI [− 0.189, − 0.070], *p* < 0.001), dysfunctional support networks (DSN) (β = 0.096, 95% CI [0.043, 0.149], *p* = 0.001), and curricular support (CS) (β = −0.052, 95% CI [− 0.092, − 0.012], *p* = 0.011) showed significant indirect effects on dropout intention. In contrast, academic self-efficacy (ASE) and labor market integration (LMI) did not exhibit significant mediation effects through this pathway.

**Table 5:** Standardized indirect effects of personal, relational, and institutional factors on university dropout intention via satisfaction with education (SE) and academic burnout (AB) (*n* = 320)

Effect type	Pathway	β	95% CI	*p*
Indirect	ASE → SE → UDI	0.012	−0.023, 0.048	0.494
	VDM → SE → UDI	0.036	0.001, 0.071	**0.042**
	FSN → SE → UDI	−0.129	−0.189, − 0.070	**< 0.001**
	DSN → SE → UDI	0.096	0.043, 0.149	**0.001**
	CS → SE → UDI	−0.052	−0.092, − 0.012	**0.011**
	LMI → SE → UDI	−0.016	−0.052, 0.020	0.375
	ASE → AB → UDI	0.246	0.143, 0.349	**< 0.001**
	VDM → AB → UDI	0.050	−0.067, 0.167	0.400
	FSN → AB → UDI	0.040	−0.050, 0.131	0.380
	DSN → AB → UDI	0.177	0.049, 0.305	**0.007**
	CS → AB → UDI	−0.061	−0.180, 0.058	0.319
	LMI → AB → UDI	0.213	0.097, 0.329	**< 0.001**
Total (direct + indirect)	ASE	0.045	−0.075, 0.164	0.460
	VDM	0.263	0.132, 0.394	**< 0.001**
	FSN	0.090	0.001, 0.179	**0.049**
	DSN	0.442	0.306, 0.579	**< 0.001**
	CS	−0.280	−0.430, − 0.130	**< 0.001**
	LMI	0.164	0.035, 0.292	**0.013**

*ASE* Academic self-efficacy, *VDM* Vocational decision-making, *FSN* Functional support networks, *DSN* Dysfunctional support networks, *CS* Curricular support, *LMI* Labor market integration, *SE* Satisfaction with education, *AB* Academic burnout, *UDI* University dropout intention, *β* standardized regression coefficient, *CI* confidence interval. Indirect effects represent mediation pathways through SE and AB. Total effects represent the sum of direct and indirect effects

Bold values indicate statistically significant coefficients (*p* < .05)

With respect to mediations via academic burnout (AB), academic self-efficacy (ASE) (β = 0.246, 95% CI [0.143, 0.349], *p* < 0.001), dysfunctional support networks (DSN) (β = 0.177, 95% CI [0.049, 0.305], *p* = 0.007), and labor market integration (LMI) (β = 0.213, 95% CI [0.097, 0.329], *p* < 0.001) showed significant indirect effects on dropout intention. By contrast, vocational decision-making (VDM), functional support networks (FSN), and curricular support (CS) did not present significant mediation effects through academic burnout (AB).

When considering the total effects (direct + indirect) on university dropout intention (UDI), significant effects were observed for vocational decision-making (VDM) (β = 0.263, 95% CI [0.132, 0.394], *p* < 0.001), functional support networks (FSN) (β = 0.090, 95% CI [0.001, 0.179], *p* = 0.049), dysfunctional support networks (DSN) (β = 0.442, 95% CI [0.306, 0.579], *p* < 0.001), curricular support (CS) (β = −0.280, 95% CI [− 0.430, − 0.130], *p* < 0.001), and labor market integration (LMI) (β = 0.164, 95% CI [0.035, 0.292], *p* = 0.013). Academic self-efficacy (ASE) did not show a significant total effect on dropout intention.

Overall, academic burnout (AB) showed the strongest direct association with university dropout intention (UDI), whereas satisfaction with education (SE) demonstrated a significant negative association of smaller magnitude. Regarding indirect effects, academic burnout (AB) mediated the associations of academic self-efficacy, dysfunctional support networks, and labor market integration with university dropout intention (UDI), while satisfaction with education (SE) mediated the effects of vocational decision-making (VDM), functional and dysfunctional support networks, and curricular support (CS).

Finally, the coefficients of determination (R²) indicated that the model explained 44.6% of the variance in satisfaction with education (SE), 34.5% of the variance in academic burnout (AB), and 86.3% of the variance in university dropout intention (UDI), demonstrating a high explanatory capacity of the model, particularly for the outcome variable.

## Discussion

The present study aimed to evaluate the empirical coherence of a structural equation model designed to explain university dropout intention (UDI) among health sciences students, incorporating personal, relational, and institutional factors, as well as the mediating role of academic burnout (AB) and satisfaction with education (SE). The adequate fit of the structural model supports the theoretical coherence of the proposed relationships and their consistency with Bronfenbrenner’s Ecological Theory of Human Development [12], demonstrating how multiple levels of the educational environment interact to influence students’ dropout intention through both direct and mediated pathways.

The findings provide robust evidence for understanding university dropout intention among health sciences students as a multidimensional and mediated phenomenon, in which personal, relational, and institutional factors converge through central academic processes such as satisfaction with education (SE) and academic burnout (AB). Consistent with the hypothesized model, the results confirm that these variables do not operate in isolation, but rather exert differential and complementary effects on dropout intention. The directionality of these associations is consistent with the operationalization of CDUe dimensions, where higher scores reflect greater risk conditions rather than protective factors.

In the present model, satisfaction with education (SE) emerged as a relevant mediating mechanism for a specific set of risk factors, particularly vocational decision-making (VDM), functional support networks (FSN), dysfunctional support networks (DSN), and curricular support (CS). The significance of these mediation effects suggests that satisfaction with education (SE) operates as a proximal process through which students integrate the perceived coherence between their vocational choice, the quality of available relational support, and the curricular conditions provided by the institution.

From this perspective, lower levels of satisfaction with education (SE) may be associated with misalignment in vocational decision-making, deficits in support networks, or limitations in curricular support, which in turn translate into a higher likelihood of university dropout intention, reinforcing the central role of the educational experience in student retention.

These findings are consistent with recent synthesized evidence [23], which identifies satisfaction with education (SE) as one of the most robust and consistent predictors of university dropout across diverse academic contexts. Complementarily, studies based on path models have shown that satisfaction with education (SE) occupies a central position within the network of variables associated with dropout, mediating the effects of academic and motivational factors on university dropout intention (UDI) [24].

Likewise, the relevance of curricular support (CS) and support networks in shaping satisfaction with education (SE) aligns with prior research highlighting the influence of institutional and organizational variables on students’ attitudes and behaviors [15, 17]. In particular, previous studies have documented that perceived quality of educational services, institutional image, and processes of organizational identification indirectly influence student retention through their impact on satisfaction with the educational experience [25–27].

In the proposed model, academic burnout (AB) was consolidated as a key mediating mechanism for a specific set of risk factors, particularly academic self-efficacy (ASE), dysfunctional support networks (DSN), and labor market integration (LMI). The significance of these mediation effects suggests that these factors are associated with university dropout intention (UDI) primarily through a process of progressive academic exhaustion, associated with a perceived imbalance between academic demands and available resources.

From this perspective, lower levels of academic self-efficacy, exposure to dysfunctional relational networks, and tensions related to labor market prospects are associated with higher levels of academic burnout (AB), thereby increasing the likelihood of considering withdrawal from university studies. These findings may reflect contextual characteristics specific to health sciences programs, which involve high academic workload, sustained training demands, and strong vocational expectations. These conditions may increase academic involvement but also heighten vulnerability to academic burnout and dropout intention.

The magnitude of the academic burnout (AB) → university dropout intention (UDI) association (β = 0.853) warrants careful consideration. Although conceptually related, academic burnout (AB) and university dropout intention (UDI) represent theoretically distinct constructs: burnout reflects a state of academic exhaustion, whereas dropout intention refers to a cognitive decision process regarding academic withdrawal. The satisfactory discriminant validity observed in the measurement models (HTMT < 0.85) supports their empirical differentiation. Therefore, the strength of the association likely reflects the central role of academic exhaustion as a proximal determinant of dropout intention in high-demand health sciences programs rather than conceptual redundancy.

These findings are consistent with empirical evidence identifying academic burnout (AB) as a central factor in the university experience of health sciences students [5, 21]. Recent studies have documented consistent associations between adverse academic conditions and higher levels of burnout among university students, particularly in high-demand programs such as those in the health sciences [4, 19]. Specifically, research conducted among nursing and medical students has shown that academic burnout (AB) is associated with both higher levels of psychological distress and a greater intention to discontinue studies, reinforcing its role as an explanatory mechanism in university dropout [5, 10]. Recent structural equation modeling studies have further confirmed the mediating role of academic burnout in the relationship between academic conditions and dropout intention, highlighting its role as a key explanatory mechanism in student persistence [41, 42].

Moreover, the mediation of academic burnout (AB) in the relationship between dysfunctional support networks (DSN) and university dropout intention (UDI) is consistent with studies highlighting the association of the relational environment on the regulation of academic stress and engagement with training [16, 20]. In this regard, interventions aimed at collaborative learning and strengthening peer support have demonstrated indirect effects on reducing academic burnout (AB), particularly among medical students [21]. Conversely, the relevance of labor market integration (LMI) as a factor associated with academic burnout (AB) aligns with evidence indicating that concerns about future employability and projected working conditions contribute to academic exhaustion and dropout intention, even prior to the completion of studies [20, 22].

The model explained a substantial proportion of the variance in university dropout intention (UDI) (R² = 86.3%), as well as a moderate proportion of the variance in satisfaction with education (SE) (R² = 44.6%) and academic burnout (AB) (R² = 34.5%). Importantly, both the CDUe and the Screening Instrument for Students At-Risk of Dropping Out demonstrated adequate evidence of validity in the present sample, supporting their appropriateness for assessing the constructs included in the model and reinforcing confidence in the interpretation of the structural relationships identified.

When the model is considered as a whole, the high explanatory power achieved for dropout intention reinforces the relevance of an integrative approach that combines personal, relational, and institutional factors with central academic mediators. These findings suggest that decisions regarding student persistence are shaped at the intersection of students’ subjective experiences and the characteristics of the educational context in which those experiences occur. Overall, the results contribute to a more integrated understanding of university dropout and underscore the need for institutional interventions that strengthen educational environments as spaces for academic development and well-being.

Overall, the results provide empirical support for the proposed hypotheses. Consistent with Hypothesis 1, personal, relational, and institutional factors showed significant associations with university dropout intention. Supporting Hypothesis 2, these factors were significantly associated with academic burnout and satisfaction with education. Finally, in line with Hypothesis 3, academic burnout and satisfaction with education functioned as key mediating mechanisms linking contextual risk factors with dropout intention. These findings reinforce the theoretical coherence of the ecological model and confirm the central role of academic experience in shaping students’ persistence decisions.

### Practical implications and limitations

The findings of this study have important implications for institutional strategies aimed at preventing university dropout among health sciences students. Rather than focusing exclusively on individual-level interventions, the results highlight the importance of addressing institutional and relational conditions that shape students’ academic experiences.

Specifically, institutional strategies could include the implementation of early monitoring systems to identify students at risk of academic burnout (AB), the strengthening of academic advising and vocational guidance programs, and the promotion of supportive academic environments that enhance students’ satisfaction with their educational experience. In addition, interventions aimed at improving curricular organization, reinforcing academic support networks, and facilitating clearer pathways toward labor market integration may contribute to reducing dropout intention.

By targeting both academic well-being and students’ perceptions of institutional support, universities may strengthen protective mechanisms that promote persistence and academic engagement in health sciences programs.

Nevertheless, the results should be interpreted in light of several limitations. First, the cross-sectional design precludes establishing causal relationships among the analyzed variables; therefore, the observed associations and mediation effects should be understood in correlational terms. Future longitudinal studies would allow for the examination of the directionality of these relationships and the assessment of the temporal stability of the identified effects.

Second, although stratified sampling with simple random selection within each stratum was employed, the study was conducted at a single institution, which may limit the generalizability of the findings to other university contexts or regions of the country. Future research should include multicenter samples encompassing institutions with diverse organizational and contextual characteristics.

Third, all variables were measured using self-report instruments, which may increase the risk of common method variance (CMV) due to the use of a single data source. Although Harman’s single-factor test did not indicate the presence of a single dominant factor accounting for the majority of the variance, this procedure is considered a limited diagnostic tool for detecting method bias [43]. Therefore, CMV cannot be entirely ruled out, and the exclusive reliance on self-reports remains a limitation of the study. In addition, the exclusive use of quantitative self-report measures limits contextual depth. Future research should incorporate multi-source data, objective academic indicators, administrative records of actual dropout, longitudinal designs to strengthen causal inferences, and qualitative approaches to triangulate findings and provide a more comprehensive understanding of dropout intention.

Finally, although the model included theoretically relevant variables, other contextual, familial, or institutional factors not considered may also influence dropout intention. Future studies could expand the model by incorporating additional variables and exploring alternative mediating or moderating mechanisms to further elucidate the complexity of student persistence processes in the health sciences.

## Conclusion

This study provides empirical evidence supporting an explanatory model of university dropout intention (UDI) among health sciences students, in which personal, relational, and institutional factors operate through two differentiated mediating mechanisms: satisfaction with education (SE) and academic burnout (AB). The findings confirm that both processes represent complementary pathways through which the educational experience is associated with decisions related to student persistence.

The identification of differentiated risk profiles, reflected in the variability of academic and relational experiences, underscores the need for analytical and strategic approaches that acknowledge student heterogeneity. In this sense, university dropout cannot be effectively addressed through uniform interventions, but rather through institutional actions that are sensitive to students’ academic trajectories and contextual conditions.

From an applied perspective, the findings suggest that dropout prevention strategies in health sciences programs should integrate interventions aimed at strengthening satisfaction with education (SE)—through curricular support (CS), vocational decision-making (VDM) guidance, and the reinforcement of functional support networks (FSN)—with actions designed to prevent and mitigate academic burnout (AB), such as academic workload management, psychoeducational support, and the promotion of student well-being. Taken together, this integrated approach offers a robust framework for the design of institutional policies and practices aimed at improving student retention and academic well-being in health sciences education.

## Supplementary Information

Supplementary Material 1.

## Data Availability

The datasets generated during the current study are not publicly available due to protect the participants’ privacy but are available from the corresponding author on reasonable request.
