# Supplementary material for: Factors associated with university dropout intention among health sciences students: the mediating role of academic burnout and satisfaction with education
Source: BMC Med Educ. 2026 Mar 9;26:622. doi: 10.1186/s12909-026-08973-7 (PMC13085532; doi:10.1186/s12909-026-08973-7)
Supplement: Supplementary file 1 — Supplementary Material 1. [file 12909_2026_8973_MOESM1_ESM.docx]

**Supplementary Material S1. Measurement Model Results**

Supplementary Table S1. Standardized Factor Loadings, Standard Errors, p Values, and Residual Variances for the CDUe Instrument (n = 320).

| **Factor - Item** | **Factor loadings** | **Standard error** | **p value** | **Residual variance** |
| --- | --- | --- | --- | --- |
| ASE Item 1 | 0.624 | — | — | 0.611 |
| ASE Item 2 | 0.537 | 0.082 | < .001 | 0.712 |
| ASE Item 3 | 0.732 | 0.090 | < .001 | 0.464 |
| ASE Item 4 | 0.631 | 0.076 | < .001 | 0.602 |
| ASE Item 5 | 0.694 | 0.082 | < .001 | 0.518 |
| ASE Item 6 | 0.692 | 0.095 | < .001 | 0.521 |
| ASE Item 7 | 0.671 | 0.092 | < .001 | 0.550 |
| ASE Item 8 | 0.829 | 0.093 | < .001 | 0.313 |
| ASE Item 9 | 0.808 | 0.087 | < .001 | 0.347 |
| ASE Item 10 | 0.817 | 0.098 | < .001 | 0.332 |
| ASE Item 11 | 0.807 | 0.090 | < .001 | 0.349 |
| ASE Item 12 | 0.748 | 0.084 | < .001 | 0.440 |
| VDM Item 1 | 0.889 | — | — | 0.210 |
| VDM Item 2 | 0.891 | 0.030 | < .001 | 0.207 |
| VDM Item 3 | 0.854 | 0.031 | < .001 | 0.270 |
| VDM Item 4 | 0.798 | 0.029 | < .001 | 0.363 |
| VDM Item 5 | 0.911 | 0.024 | < .001 | 0.169 |
| VDM Item 6 | 0.917 | 0.022 | < .001 | 0.159 |
| VDM Item 7 | 0.936 | 0.022 | < .001 | 0.124 |
| FSN Item 1 | 0.674 | — | — | 0.546 |
| FSN Item 2 | 0.781 | 0.070 | < .001 | 0.390 |
| FSN Item 3 | 0.772 | 0.060 | < .001 | 0.404 |
| FSN Item 4 | 0.790 | 0.066 | < .001 | 0.376 |
| FSN Item 5 | 0.736 | 0.061 | < .001 | 0.459 |
| FSN Item 6 | 0.759 | 0.060 | < .001 | 0.424 |
| FSN Item 7 | 0.569 | 0.063 | < .001 | 0.677 |
| FSN Item 8 | 0.519 | 0.061 | < .001 | 0.731 |
| FSN Item 9 | 0.930 | 0.076 | < .001 | 0.136 |
| FSN Item 10 | 0.905 | 0.077 | < .001 | 0.180 |
| DSN Item 1 | 0.641 | — | — | 0.590 |
| DSN Item 2 | 0.748 | 0.082 | < .001 | 0.441 |
| DSN Item 3 | 0.783 | 0.092 | < .001 | 0.387 |
| DSN Item 4 | 0.752 | 0.088 | < .001 | 0.434 |
| DSN Item 5 | 0.743 | 0.091 | < .001 | 0.448 |
| DSN Item 6 | 0.795 | 0.090 | < .001 | 0.368 |
| DSN Item 7 | 0.910 | 0.101 | < .001 | 0.171 |
| DSN Item 8 | 0.881 | 0.098 | < .001 | 0.224 |
| CS Item 1 | 0.685 | — | — | 0.531 |
| CS Item 2 | 0.800 | 0.053 | < .001 | 0.360 |
| CS Item 3 | 0.700 | 0.056 | < .001 | 0.510 |
| CS Item 4 | 0.655 | 0.054 | < .001 | 0.572 |
| CS Item 5 | 0.776 | 0.058 | < .001 | 0.398 |
| CS Item 6 | 0.763 | 0.061 | < .001 | 0.418 |
| CS Item 7 | 0.758 | 0.050 | < .001 | 0.426 |
| CS Item 8 | 0.600 | 0.063 | < .001 | 0.640 |
| CS Item 9 | 0.754 | 0.060 | < .001 | 0.431 |
| CS Item 10 | 0.779 | 0.059 | < .001 | 0.394 |
| CS Item 11 | 0.690 | 0.063 | < .001 | 0.523 |
| CS Item 12 | 0.726 | 0.060 | < .001 | 0.472 |
| LMI Item 1 | 0.729 | — | — | 0.468 |
| LMI Item 2 | 0.814 | 0.034 | < .001 | 0.337 |
| LMI Item 3 | 0.835 | 0.037 | < .001 | 0.303 |
| LMI Item 4 | 0.862 | 0.040 | < .001 | 0.257 |
| LMI Item 5 | 0.821 | 0.040 | < .001 | 0.325 |
| LMI Item 6 | 0.859 | 0.047 | < .001 | 0.262 |
| LMI Item 7 | 0.833 | 0.043 | < .001 | 0.307 |
| LMI Item 8 | 0.763 | 0.046 | < .001 | 0.417 |
| LMI Item 9 | 0.842 | 0.044 | < .001 | 0.292 |
| LMI Item 10 | 0.732 | 0.042 | < .001 | 0.464 |
| LMI Item 11 | 0.790 | 0.041 | < .001 | 0.375 |
| LMI Item 12 | 0.757 | 0.043 | < .001 | 0.427 |
| LMI Item 13 | 0.877 | 0.043 | < .001 | 0.232 |
| LMI Item 14 | 0.797 | 0.039 | < .001 | 0.364 |
| Note. ASE = Academic self-efficacy; VDM = Vocational decision-making; FSN = Functional support networks; DSN = Dysfunctional support networks; CS = Curricular support; LMI = Labor market integration. CDUe: University Dropout Questionnaire for Students. The first loading of each factor was fixed to scale the latent variable; therefore, no standard error or p value is reported for those parameters. | | | | |

Supplementary Table S2. Discriminant Validity of CDU Latent Constructs Assessed via the Heterotrait–Monotrait Ratio (HTMT) (n = 320).

| **Construct** | **1** | **2** | **3** | **4** | **5** | **6** |
| --- | --- | --- | --- | --- | --- | --- |
| 1.ASE | 1 |  |  |  |  |  |
| 2.VDM | 0.560 | 1 |  |  |  |  |
| 3.FSN | 0.202 | 0.225 | 1 |  |  |  |
| 4.DSN | 0.532 | 0.567 | 0.218 | 1 |  |  |
| 5.CS | 0.544 | 0.501 | 0.126 | 0.600 | 1 |  |
| 6.LMI | 0.558 | 0.620 | 0.184 | 0.517 | 0.567 | 1 |
| Note. ASE = Academic self-efficacy; VDM = Vocational decision-making; FSN = Functional support networks; DSN = Dysfunctional support networks; CS = Curricular support; LMI = Labor market integration. CDUe: University Dropout Questionnaire for Students. Values represent HTMT ratios. Values below 0.85 indicate adequate discriminant validity. | | | | | | |

Supplementary Table S3. Standardized Factor Loadings, Standard Errors, p Values, and Residual Variances for the Screening Instrument for Students At-Risk of Dropping Out (n = 320).

| **Factor Item** | **Factor loadings** | **Standard error** | **p value** | **Residual variance** |
| --- | --- | --- | --- | --- |
| SE Item 1 | 0.909 | — | — | 0.173 |
| SE Item 2 | 0.965 | 0.015 | < .001 | 0.068 |
| SE Item 3 | 0.917 | 0.015 | < .001 | 0.159 |
| SE Item 4 | 0.939 | 0.015 | < .001 | 0.119 |
| UDI Item 1 | 0.784 | — | — | 0.386 |
| UDI Item 2 | 0.880 | 0.035 | < .001 | 0.226 |
| UDI Item 3 | 0.956 | 0.036 | < .001 | 0.086 |
| UDI Item 4 | 0.976 | 0.038 | < .001 | 0.047 |
| AB Item 1 | 0.761 | — | — | 0.421 |
| AB Item 2 | 0.898 | 0.035 | < .001 | 0.193 |
| AB Item 3 | 0.855 | 0.031 | < .001 | 0.269 |
| AB Item 4 | 0.930 | 0.035 | < .001 | 0.136 |
| Note. SE = Satisfaction with Education; UDI = University Dropout Intention; AB = Academic Burnout. The first loading of each factor was fixed to scale the latent variable; therefore, no standard error or p value is reported for those parameters. | | | | |

Supplementary Table S4. Discriminant Validity of Dropout Risk Latent Constructs Assessed via the Heterotrait–Monotrait Ratio (HTMT) (n = 320).

| **Construct** | **1** | **2** | **3** |
| --- | --- | --- | --- |
| 1.SE | 1 |  |  |
| 2.UDI | 0.300 | 1 |  |
| 3.AB | 0.124 | 0.825 | 1 |
| Note. SE = Satisfaction with education; UDI = University dropout intention; AB = Academic burnout. Values represent HTMT ratios. Values below 0.85 indicate acceptable discriminant validity. | | | |

Supplementary Table S5. Correlations Among Exogenous Latent Variables in the Final Structural Model (n = 320).

| **Variable** | **1** | **2** | **3** | **4** | **5** | **6** |
| --- | --- | --- | --- | --- | --- | --- |
| 1. ASE | 1 |  |  |  |  |  |
| 2. VDM | .544** | 1 |  |  |  |  |
| 3. FSN | −.106** | −.183** | 1 |  |  |  |
| 4. DSN | .532** | .551** | −.124** | 1 |  |  |
| 5. CS | .541** | .487** | −.047** | .600** | 1 |  |
| 6. LMI | .561** | .624** | −.144** | .524** | .560** | 1 |
| Notes. ASE = Academic self-efficacy; VDM = Vocational decision-making; FSN = Functional support networks; DSN = Dysfunctional support networks; CS = Curricular support; LMI = Labor market integration; SE = Satisfaction with education; AB = Academic burnout; UDI = University dropout intention. Values represent standardized correlations among latent variables.* *p* < 0.05; ** *p* < 0.001. | | | | | | |
